# Probiotic potential characterization and clustering using unsupervised algorithms of lactic acid bacteria from saltwater fish samples

**DOI:** 10.1038/s41598-022-16322-z

**Published:** 2022-07-13

**Authors:** Atefeh Mazlumi, Bahman Panahi, Mohammad Amin Hejazi, Yousef Nami

**Affiliations:** 1 Faculty of Agriculture, Tabriz University, Tabriz, Iran; 2 Department of Genomics, Branch for Northwest & West region, Agricultural Biotechnology Research, Institute of Iran, Agricultural Research, Education and Extension Organization (AREEO), Tabriz, Iran; 3 Department of Food Biotechnology, Branch for Northwest & West region, Agricultural Biotechnology Research, Institute of Iran, Agricultural Research, Education and Extension Organization (AREEO), Tabriz, Iran

**Keywords:** Biotechnology, Microbiology

## Abstract

This research aimed to isolate lactic acid bacteria from the bowel of saltwater fish to assess their potential probiotic properties. Nineteen isolates of LAB including *Lactiplantibacillus plantarum*, *Lactiplantibacillus pentosus*, *Lactobacillus acidophilus*, *Levilactobacillus brevis*, *Pediococcus pentosaceus*, and *Pediococcus acidilactici* were recognized using molecular tools. All the isolates survived in the simulated conditions of the GI tract. Auto-aggregation ranged from 01.3 ± 0.5 to 82.6 ± 1.4% and hydrophobicity with toluene ranged from 3.7 ± 1.6 to 69.4 ± 1.3%, while the range of hydrophobicity with xylene was from 02.2 ± 1.6 to 56.4 ± 2.1%. All the isolates of lactobacilli, pediococci, enterococci, and lactococci indicated variable sensitivity and resistance towards clinical antibiotics. Non-neutralized cell free supernatant of isolates F12 and F15 showed antimicrobial activity against all the 8 evaluated enteric pathogens. Cluster analysis of identified potential probiotic bacteria based on heat-map and PCA methods also highlighted the priority of isolates F3, F7, F12, and F15 as bio-control agents in fishery industry. The findings of this study may essentially contribute to the understanding of the probiotic potential of LAB in saltwater fish, in order to access their probiotic characterization for use as biocontrol in fishery.

## Introduction

Aquaculture is a significant food sector for a growing global human people and has quickly extended due to intensified culture methods^1^. Nonetheless, the expansion, diversity and enhancement of aquaculture have increased the frequency of disease epidemics in recent decades^2^, with bacteria being the largest pathogen in aquaculture and causing mass mortality in aquaculture^3^. Handwork of intestinal biota utilizing dietary supplements is an inventive confronts to correct the improvement capability and intestine security of lentic life forms^4^. It has been suggested that pathogenic organisms in the animal's gastrointestinal tract may disrupt the immune systems, and cause unfavorable colonialism and digestibility issues. Therefore, screening and selection of proper probiotic against these pathogenic organisms is focused to utilize these bacteria in the aquaculture industry^5^.

The application of probiotics as one of the useful feed additives have a significant impact on disease resistance, growth, immune response, and other beneficial effects on the culture host. It has been shown that the use probiotics increases resistance to diseases of fish and shrimp, strengthens immunity and improves water quality^6^. Moreover, as an environmentally friendly strategy, probiotics have been proposed as a means for combating disease outbreaks and preventative treatments. Intensive farming practices, with the aim of increasing production and maximizing profits, have contributed to an increase in the problem of diseases caused by infections by viruses, bacteria, fungi, parasites and other pathogens^7^.

The broad antibacterial spectrum of probiotics and their strong antibacterial activity against pathogens are their important functional properties^8^. In addition to working on the principle of exclusion of competitors and using immune-stimulators to combat illness, probiotic bacteria produce digestive enzymes that improve digestion, thereby improving host stress tolerance and health^6^. Probiotics generally exhibit antibacterial, antiviral, and antifungal properties^9^. The application of probiotics in aquaculture extends to a wide variety of hosts, including fish and shellfish, which provide health benefits^10^.

The generic probiotic microorganism should able to increase and colonize in the host gut. The association of gut microbiota modulation with physiological and health status is increasing consideration in fish industry^11^. Probiotics used in terrestrial animals may not be able to colonize the intestines of aquaculture organisms^12^. On the other hand, the fish gut flora is digestible, maintains mucosal resistance, a vigorous host immune response, and provides some protection against gastric infections, thus playing an important role in mediating and agitating the host's gastrointestinal dilatation^13,14^.

Among different lactic acid bacteria (LAB), the probiotic effects on fish were confirmed for *Lactobacillus pentosus*^15^, *Lactobacillus paraplantarum*^16^, *Enterococcus faecium*^17^, *Enterococcus faecalis*^18^, *Pediococcus pentosaceus*^19^, and *Lactococcus lactis*^20^. However, identification and characterization of new strains with desired features are inevitable^21^. In this work, bacteria colonized in the bowel of fish were isolated and characterized as potential probiotic bacteria. Then, the potential of characterized bacteria as a biocontrol agent in fish industry were surveyed.

## Materials and methods

### LAB strains and culture condition

A total of 15 fresh edible saltwater fish samples including Silver Pomfret (*Pampus argenteus*)*,* Tigertooth Croaker (*Otolithes ruber*), King Fish (*Scomberomorus commerson*), Seer fish (*Scomberomorus guttatus*) and Thornycheek Grouper (*Epinephelus diacanthus*), weighing 500 ± 50 g were purchased from retail fish shops in Qeshm Island (located in Persian Gulf, South of Iran). The samples were transferred on ice to the laboratory of Gol Sahand Khosrou Science-based Company. To isolate LAB from the intestinal contents of the fish samples, 5 g of intestinal contents was inoculated into 50 mL of de Man Rogosa and Sharpe (MRS, Merck, Darmstadt, Germany) broth containing 25 g/L NaCl (DRM CHEM, Arak, Iran) and incubated at 37 °C for 3–7 days. Then, all cultures were streaked separately on MRS agar plate and incubated anaerobically at 37 °C for 3–7 days in anaerobic jars containing anaerobic gas generation kits. Colonies with different morphology were isolated from the incubated MRS agar plates. After morphological and biochemical assays (Gram-staining, catalase activity, cell morphology, and spore formation), Gram-positive and catalase-negative rods- and cocci-shaped bacteria were identified as LAB isolates.

### Acid and bile resistance

The MRS broth with pepsin enzyme (3 mg/mL) was utilized as a medium to assess acid resistance. The pH of broth was adjusted to 2.5 with 1.0 N HCl and broth (pH 7.0) was used as a control. Moreover, the broth was inoculated for 3 h and the optical density (OD) was measured at 600 nm. The acid resistance was determined by the following equation: Survival Rate (%): [OD (After treatment)/OD (Before treatment)] × 100%. Strains with viability more than 80% were selected for further analysis.

The ability of isolates to grow in presence of bile salt was measured. This was carried out with 0.3% w/v oxgall and Control was maintained employing MRS broth. The samples were inoculated at 37 °C for 4 h and OD_620_ of samples was measured to check the viability of cells. The oxgall resistance was determined by the following equation: Survival Rate (%): [OD (After treatment)/OD (Before treatment)] × 100%.

The viability of selected strains in simulated gastrointestinal conditions was measured based on method described by Nami et al. (2019)^17^. For this purpose, cells cultured overnight were collected by centrifugation at 1500 × g at 4 °C for 15 min. The cell pellet was washed twice with neutralized PBS solution and resuspended in simulated gastric juice (SGF) and simulated intestinal juice (SIF) with 10^8^ colony forming units CFU ml^−1^. Sterilized PBS (pH 3) supplemented with pepsin (3 mg ml^−1^) and sterile PBS (pH 8) supplemented with SGF and pancreatin (1 g L^−1^, Sigma Aldrich, St. Louis, Missouri).) Was prepared., USA) and bovine bile (0.3 g L^−1^) were used to prepare SIF. The resistance profile was examined by counting colonies on the MRS agar plate after 0, 1, 2, 3 h for SGF and after 0, 1, 2, 3, 4 h for SIF experiments.

### Antimicrobial activity

The antimicrobial features of isolates were performed by agar well diffusion assay. In this method, plates containing Mueller–Hinton agar medium impregnated with different indicator bacteria was used (Table 1). A sterile pasteurized pipette was used to create wells in medium. Finally, 100 µL of supernatants of isolates were placed inside each wells and plates were then incubated at 37 °C for 24 h. After incubation time, the measured inhibition halo zone diameters were statistically analyzed^22^.

**Table 1:** Zone inhibition (mm) of antimicrobial properties of isolates.

Isolates	Indicator bacteria							
	*Vibrio harveyi*	*Vibrio cholera*	*Streptococcus iniae*	*Vibrio alginolyticus*	*Vibrio fluvialis*	*Vibrio parahaemolyticus*	*Streptococcus agalactia*	*Clostridium cochlearium*
F1	0	17	14	17	19	0	21	22
F2	8	0	21	20	0	17	19	13
F3	14	18	10	0	16	13	22	20
F4	0	15	17	9	21	16	16	19
F5	16	0	11	12	9	18	13	14
F6	11	13	20	8	0	21	17	14
F7	22	21	19	0	12	0	10	18
F8	13	11	11	11	17	12	18	0
F9	0	0	16	15	19	9	22	14
F10	9	10	14	0	0	8	15	21
F11	0	0	22	0	0	0	24	0
F12	19	22	28	23	19	21	31	26
F13	8	9	12	0	12	15	16	0
F14	0	0	20	0	0	0	19	0
F15	21	19	26	22	17	14	29	25
F16	0	7	16	19	8	19	19	18
F17	14	11	19	10	0	16	21	12
F18	0	0	24	0	0	0	17	0
F19	12	8	21	17	18	0	12	0

### Antibiotic susceptibility

The disc diffusion method was performed for evaluation of the susceptibility of isolates to some high-consumption and clinically important antibiotics listed in Table 2. For this purpose, isolates were streaked over the solidified MRS medium and antibiotic disks were placed on the medium and incubated overnight at 37 °C. Finally, the diameters of inhibition zone around disks were measured by digital caliper^23^.

**Table 2:** Antibiotic susceptibility profile of isolates.

Isolates	Antibiotics									
	Ampicillin (10 µg)	Vancomycin (30 µg)	Erythromycin (15 µg)	Azithromycin (15 µg)	Gentamycin (10 µg)	Penicillin (10 µg)	Chloramphenicol (30 µg)	Tetracycline (30 µg)	Streptomycin (10 µg)	Ciprofloxacin (5 µg)
F1	I	R	S	R	I	R	S	S	S	I
F2	R	I	R	I	I	I	S	S	R	R
F3	S	S	S	S	S	S	S	S	S	S
F4	S	R	I	I	R	R	R	I	R	I
F5	S	I	S	R	R	R	S	S	I	R
F6	S	R	S	I	R	R	S	I	R	I
F7	S	S	S	S	S	S	S	S	S	S
F8	S	R	S	R	S	I	S	S	I	R
F9	S	I	S	R	R	R	S	S	R	I
F10	S	S	S	S	R	S	S	S	R	R
F11	R	R	I	R	R	R	S	S	I	R
F12	S	S	S	S	S	S	S	S	S	S
F13	S	R	S	S	R	R	S	S	R	R
F14	S	I	S	R	R	R	S	S	I	R
F15	S	S	S	S	S	S	S	S	S	S
F16	I	R	S	R	I	R	S	S	R	R
F17	S	I	R	R	R	R	S	I	S	R
F18	R	R	R	R	R	R	R	R	R	R
F19	S	R	S	S	R	S	S	S	I	R

I: intermediate susceptibility (zone diameter 12.5–17.4 mm); R: resistant (zone diameter < 12.4 mm); S: susceptible (zone diameter > 17.5).

### Cell surface hydrophobicity

The adhesion ability of isolates to xylene and toluene was determined as previously described by^24^. The test was performed in triplicate and expressed as:

$$ {\text{Hydrophobicity }}\left( \% \right) \, = \, \left( {{1 } - {\text{ A1}}/{\text{A}}0} \right) \, \times { 1}00. $$

where A_0_ represents the initial absorbance at 600 nm before adding xylene and toluene; A_1_ represents absorbance after 4 h inoculated with xylene and toluene.

### Autoaggregation assay

The ability of isolates to auto-aggregate was performed according to the method described by^25^. Auto-aggregation percentage was determined using the following equation:

$$ {\text{Auto}} - {\text{aggregation }}\left( \% \right) \, = { 1 } - \, \left( {{\text{At}}/{\text{A}}0} \right) \, \times { 1}00 $$

where A_0_ represents absorbance at t = 0 and at represents absorbance at time t.

### Coaggregation assay

Co-aggregation of isolates against *Staphylococcus aureus* and *bacillus cereus* was performed based on method used by^26^. Co-aggregation percentage was calculated based on equation: % = A_0_ − A_t_/A_t_ × 100.

### Adhesion ability to human intestinal cells

Selected isolates were explored for their attachment capacity to the human colon carcinoma cell line Caco-2. RPMI medium supplemented with 10% heat-inactivated fetal bovine serum and 1% penicillin–streptomycin blend were utilized and cells were refined on 24-well tissue culture plates and incubated at 37 °C in 5% CO_2_ under a relatively humidified atmosphere until a confluent monolayer was formed. Sometime recently the attachment measure, the media within the wells containing a Caco-2 cell monolayer were evacuated and supplanted with new antibiotic-free RPMI. From that point, 1 × 10^7^ CFU/mL of isolates was included to each well with a add up to volume of 1 mL and after that incubated for 3 h at 37 °C under an atmosphere of 5% (v/v) CO_2_. The wells were washed twice with a sterile pre-warmed PBS solution to evacuate non-attached bacterial cells. One mL of 1% (v/v) Triton X-100 was included to each well to withdraw the cells from the wells and the blend was mixed for 10 min. To measure the viable cell count, the cell suspension was plated onto MRS agar and hatched at 37 °C^27^.

### Cholesterol assimilation

The cholesterol-removing capacity of the isolate was determined using the method previously described by Nami et al.^28^. Isolates were inoculated for 20 h into MRS broth supplemented with water-soluble cholesterol (polyoxyethanyl cholesteryl sebacate; Sigma) and 0.3% bovine bile at a concentration of 150 μg/mL at 37 °C. After the incubation period, the cells were centrifuged at 4250 × g for 15 min to obtain cell pellets, and the cholesterol remaining in the upper layer was measured by the o-phthaldehyde method.

### Biofilm formation

The ability of isolates to form biofilm was determined according to method described by^29^ with some modifications. For this purpose, the wells of a sterile 6-well tissue culture plate were filled with 5 mL of MRS broth supplemented with 500 µL of overnight isolates (10^7^–10^8^ CFU/mL). Cultures were incubated anaerobically for 48 h at 37 °C. After this period, the wells were gently washed three times with 5 mL of sterile distilled water. Then, 3 mL of methanol was used for 15 min to fix the attached bacteria and then plates were emptied and dried at room temperature. Afterwards, 3 mL of a 2% (v/v) crystal violet solution was poured in the wells and held at room temperature for 5 min. Finally, to release stain from Adherent cells, 2 mL of 33% (v/v) glacial acetic acid was used and the optical density (OD) of each well was measured at 595 nm using a platereader (Microplatereader, Bio-Rad, Hercules; CA, USA).

### Exopolysaccharide (EPS) production

To assess EPS production ability of isolates, method described by^30^ with slight modification was used. Briefly, modified MRS agar medium (replacing glucose with 100 g/L of sucrose) was used and isolates were streaked on cultures and incubated at 37 °C for 24 h. Metal loop was used to drag up formed colonies. If the length of slime was above 1.5 mm, the isolate was considered positive slimy producers.

### Molecular identification

#### Genomic DNA extraction

The total genomic DNA of the isolates was extracted through the procedure designed in our laboratory. For this purpose, a single colony of each isolate was placed in 0.2 tube and 20 µL lysis buffer was poured inside the tube. The tubes were gently vortexed until the contents were completely homogeneous and were placed for one hour at room temperature. Then, they were incubated in thermal cycler PTC 200 (MJC research, Waltham, USA) for 10 min at 85 °C. After this period, 150 µL deionized water was added to tubes and they were centrifuged at 8000 × g for 5 min. Finally, the upper phase containing genomic DNA was removed and poured in new tubes. The genomic DNA was stored at refrigerator until using.

#### Amplification of 16S-rRNA gene by polymerase chain reaction (PCR)

The amplification of genomic DNA samples of the isolates was performed in a thermal cycler PTC 200 by using a pair of primers (Hal-6F: 5'-AGAGTTTGATCMTGGCTCAG-3') and Hal-R6: 5'-TACCTTGTTAGGACTTCACC-3')^31,32^. The following temperature profile was used to amplify DNA: an initial denaturation at 95 °C for 5 min, followed by 32 cycles of denaturation at 94 °C for 60 s, annealing at 59 °C for 60 s, extension at 72 °C for 60 s, and a final extension step at 72 °C for 5 min. Electrophoresis in a 0.8% (w/v) agarose gel was used to resolve the PCR products and visualized via ethidium bromide staining.

#### 16S-rRNA gene sequencing

The PCR products of the 16S-rRNA gene (1544 bp) were amplified using the mentioned primer set. The PCR products were sequenced at Sinaclone Corporation, Tehran, Iran. The sequences were then analyzed using the BLAST program of the National Center for Biotechnology Information (http://www.ncbi.nlm.nih.gov/BLAST).

### Statistical analysis

To determine the significant differences between the parameters of each isolates (*P* ≤ *0.05*), analysis of variance (ANOVA) and Duncan's test were used. Moreover, excel 2013 (Microsoft Corporation) and SPSS (IBM SPSS Statistics 20) were used for formal statistical analysis.

### Unsupervised clustering algorithms

The heat-map was generated using Euclidean distance measure and Ward clustering methods implemented in ggplot2 package of R software as prescribe in^33^. For unsupervised clustering of samples based on desired probiotic characteristics, principal component analysis (PCA) method was used. This algorithm provides a means to achieve unbiased dimensionality reduced structure of studies strains^34^.

### Ethics statement

No ethical issues were promulgated.

## Results

### Morphological and biochemical assays

**Figure 1:**
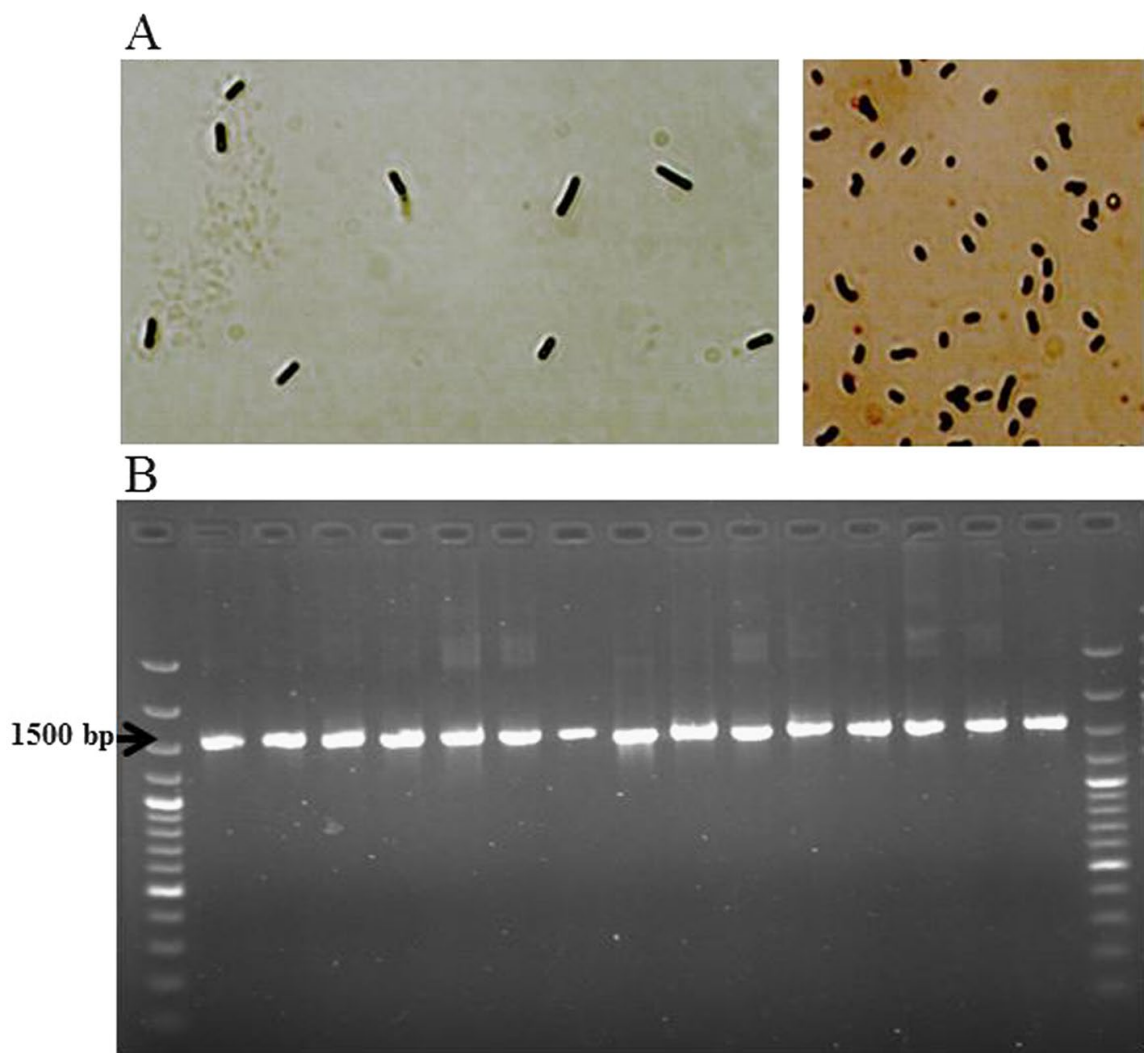
Gram-staining test (**A**) and the quality and size of 16S-rRNA gene (1544 bp) amplified by a pair of primers (Hal-6F and Hal-R6). 1 kb ladder was used (**B**).

A total of 31 rod- and cocci-shaped colonies (Fig. 1) were grown and isolated on culture media. Each colony was separately propagated for assessments. Based on Gram-positive and catalase-negative assays, 19 isolates were identified as LAB isolates and selected for further analysis.

### Acid and bile tolerance

**Figure 2:**
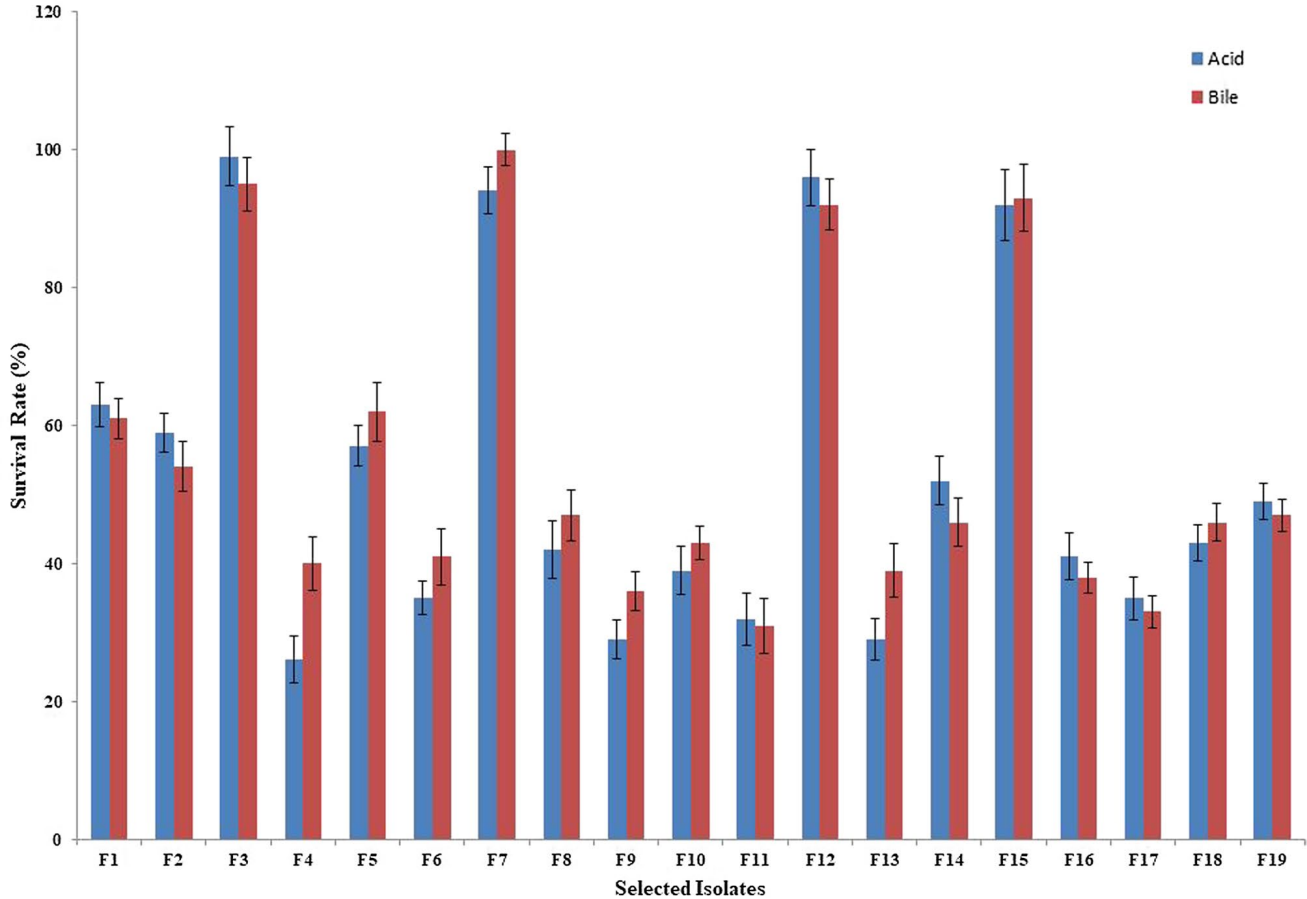
Tolerance of isolates to low pH and high bile salt concentrations.

Figure 2 represents the survival rates of 19 rod- and cocci-shaped isolates after 3 h of incubation at pH 3.0. Based on the findings, all 19 isolates retained their viability and percent survival rates of evaluated isolates ranged from 26 to 100% after 3 h of incubation at 37 °C (data not shown). The isolates with the most efficient resistance to low pH conditions were isolates F3, F12, F7, and F15 with survival rates of 99, 96, 94, and 92%, respectively. Conversely, isolates F4, F9, and F11 indicated the lowest viability after 3 h, with survival rates of 26, 29, and 32%, respectively.

The survival rates of 19 isolates in 0.3% oxgall are indicated in Fig. 2. All 19 isolates appeared resistant to high bile salt condition, despite variations in the degree of viability. Percent survival rates of evaluated isolates ranged from 31 to 100% after 4 h of incubation at 37 °C. The highest tolerance was observed for isolate F7, followed by isolates F3, F15, and F12 with survival rates of 100, 95, 93, and 92%, respectively. Isolates F11, F17, and F9 showed the lowest viability after 4 h of incubation in media containing 0.3% oxgall, with survival rates of 31, 33, and 36%, respectively.

### Antimicrobial activity

Table 1 indicates the antibacterial activity of the evaluated isolates against several Gram-positive and Gram-negative bacteria. Eight important aquatic pathogens including *Vibrio harveyi* (PTCC 1755), *Vibrio cholera* (PTCC 1611), *Streptococcus iniae* (PTCC 1887), *Vibrio alginolyticus* (ATCC 17,749), *Vibrio fluvialis* (ATCC 33,809), *Vibrio parahaemolyticus* (ATCC 17,802), *Streptococcus agalactia* (ATCC 12,386), and *Clostridium cochlearium* (PTCC 1263) were used. The neutralized supernatant fluids of isolates F15 and F12 were able to inhibit the growth of all the eight indicator bacteria. Meanwhile isolates F11, F14, and F18 were able to inhibit the growth of two Gram-positive indicators named *St. iniae* and *St. agalactia,* while the neutralized supernatant fluids of these isolates were ineffective against any of the Gram-negative pathogen tested in the work.

### Antibiotic susceptibility

The selected isolates were assessed for their antibiotic susceptibility against ciprofloxacin (5 µg), ampicillin (10 µg), vancomycin (30 µg), erythromycin (15 µg), azithromycin (15 µg), gentamicin (10 µg), penicillin (10 µg), chloramphenicol (30 µg), tetracycline (30 µg), and streptomycin (10 µg). Based on the results illustrated in Table 2, isolate F12 was susceptible to all the tested antibiotics, while isolate F18 was resistant to all the tested antibiotics. The most widespread resistance among the isolates was observed against gentamycin, penicillin, and ciprofloxacin which were detected in 57.9% of the isolates, while the least resistance was observed against tetracycline which was detected only in isolate F18.

### Cell surface hydrophobicity

The cell surface hydrophobicity percentages of the isolates ranged from 3.7 ± 1.6% to 70.2 ± 2.0% and 2.2 ± 1.6% to 76.4 ± 2.1% with toluene and xylene, respectively (Table 3). The highest hydrophobicity percentages with toluene and xylene were observed for isolates F15 (70.2 ± 2.0%) and F12 (76.4 ± 2.1%), respectively. Conversely, isolates F18 (3.7 ± 1.6%) and F11 (2.2 ± 1.6%) possessed the least hydrophobicity with toluene and xylene, respectively. These results display that hydrophobicity is a strain-dependent property and that LAB strains are naturally hydrophilic.

**Table 3:** Hydrophobicity, adhesion ability and cholesterol assimilation capacity of selected strains.

Isolates	LAB species	Hydrophobicity (%)		Auto-aggregation (%)	Co-aggregation (%)		Adhesion ability (%)	Cholesterol assimilation (%)
		Toluene	Xylene		*Staphylococcus aureus*	*Bacillus cereus*		
F1	*Lactobacillus plantarum*	30.2 ± 1.8^ cd^	29.1 ± 1.6f.	24.6 ± 1.5^i^	27.3 ± 1.7^i^	25.1 ± 1.6f.	0	19.2 ± 1.1^ k^
F2	*Pediococcus lolli*	34.9 ± 1.6^c^	34.2 ± 1.7^e^	28.4 ± 1.7^ h^	30.4 ± 2.1^gh^	29.4 ± 1.7^e^	0	23.9 ± 1.6^j^
F3	*Lactobacillus acidophilus*	69.4 ± 1.3^a^	65.6 ± 1.9^d^	75.6 ± 1.9^b^	52.4 ± 2.3^c^	61.2 ± 2.2^a^	24.7 ± 1.7	64.5 ± 1.8^b^
F4	*Pediococcus lolli*	30.4 ± 2.1^ cd^	28.8 ± 1.7^ fg^	31.4 ± 1.7f.	36.4 ± 1.9^e^	33.8 ± 1.4^d^	0	52.1 ± 1.8^d^
F5	*Lactobacillus acidophilus*	28.4 ± 1.6^ cd^	27.3 ± 1.5^gh^	33.4 ± 1.9^e^	29.7 ± 1.7^ h^	28.9 ± 2.1^e^	0	37.4 ± 1.9^ g^
F6	*Lactobacillus plantarum*	25.7 ± 1.8^ cd^	23.5 ± 1.2^i^	29.7 ± 2.2^gh^	19.4 ± 1.3^j^	17.5 ± 1.6^i^	0	33.5 ± 2.1^ h^
F7	*Pediococcus pentosaceus*	66.1 ± 1.3^a^	72.3 ± 1.7^b^	82.6 ± 1.4^a^	55.3 ± 2.1^b^	49.7 ± 1.8^c^	27.5 ± 1.9	59.7 ± 2.2^c^
F8	*Lactococcus lactis*	26.3 ± 2.1^ cd^	23.6 ± 1.5^i^	20.4 ± 1.2^j^	16.2 ± 1.7^ k^	14.9 ± 1.5^j^	31.4 ± 2.1	31.2 ± 2.1^i^
F9	*Pediococcus acidilactici*	27.1 ± 1.4^ cd^	25.8 ± 2.1^ h^	36.2 ± 2.3^d^	31.5 ± 1.9^ fg^	33.8 ± 1.4^d^	0	22.5 ± 0.9^j^
F10	*Lactococcus lactis*	30.1 ± 1.9^ cd^	29.8 ± 2.0f.	31.2 ± 2.1^ fg^	27.7 ± 1.5^i^	26.8 ± 1.4^e^	0	37.2 ± 1.8^ g^
F11	*Enterococcus hirae*	04.9 ± 1.1^ g^	02.2 ± 1.6^n^	04.3 ± 1.4^ m^	08.6 ± 1.4^i^	03.2 ± 1.6^ m^	0	11.8 ± 0.9^ l^
F12	*Lactobacillus plantarum*	66.8 ± 1.8^b^	76.4 ± 2.1^a^	73.8 ± 2.3^c^	58.3 ± 1.8^a^	53.4 ± 2.2^b^	38.7 ± 1.5	78.2 ± 1.9^a^
F13	*Pediococcus pentosaceus*	10.2 ± 1.4^ fg^	08.4 ± 1.3^ l^	10.4 ± 1.6^ k^	02.9 ± 1.4^ m^	09.4 ± 1.5^ k^	0	42.8 ± 2.6f.
F14	*Pediococcus pentosaceus*	22.9 ± 1.7^de^	22.7 ± 1.5^i^	25.7 ± 1.8^i^	20.3 ± 1.6^j^	21.2 ± 1.9^ g^	0	47.2 ± 2.2^e^
F15	*Pediococcus acidilactici*	70.2 ± 2.0^a^	68.2 ± 1.9^c^	72.4 ± 2.3^c^	42.1 ± 1.2^d^	35.5 ± 1.4^d^	23.2 ± 1.5	30.7 ± 1.7i
F16	*Lactobacillus fermentum*	14.4 ± 1.8^ef^	13.7 ± 1.4^j^	18.9 ± 1.7^j^	15.5 ± 1.6^ k^	18.2 ± 1.9^ h^	0	33.4 ± 1.9^ h^
F17	*Lactococcus lactis*	29.1 ± 1.5^ cd^	28.2 ± 1.9^ fg^	35.7 ± 1.4^d^	32.2 ± 2.3^ fg^	27.5 ± 1.1^ef^	0	29.8 ± 2.5^i^
F18	*Enterococcus hirae*	03.7 ± 1.6^ g^	05.9 ± 1.1^ m^	08.1 ± 1.7^ l^	02.5 ± 1.2^ m^	05.5 ± 1.6^ l^	0	03.6 ± 1.2^n^
F19	*Enterococcus lactis*	11.4 ± 1.8^ fg^	10.6 ± 1.8^ k^	01.3 ± 0.5^n^	03.8 ± 1.8^ m^	01.4 ± 0.9^n^	0	7.25 ± 1.3^ m^

Values are mean ± standard error.

^a–n^Values followed by the same letters are not significantly different (*P* > *0.05*). Statistical analysis of each formulation was done separately.

### Autoaggregation assay

Results of the autoaggregation of the 19 selected isolates are illustrated in Table 3. Values of autoaggregation ranged from 1.3 ± 0.5% to 82.6 ± 1.4% after 4 h of incubation. The highest autoaggregation values was observed for isolate F7, with value of 82.6 ± 1.4%, while the least value was obtained for isolate 11, with 1.3 ± 0.5% autoaggregation after 4 h of incubation.

### Coaggregation assay

Table 3 shows the results of the coaggregation ability of the 19 evaluated isolates. The coaggregation percentages ranged from 2.5 ± 1.2% to 58.3 ± 1.8% with *Staphylococcus aureus* and from 1.4 ± 0.9% to 61.2 ± 2.2% with *Bacillus cereus*. The highest coaggregation value with *S. aureus* was observed for isolate F12 (58.3 ± 1.8%), while the highest value for coaggregation with *B. cereus* was obtained for isolate F3 (61.2 ± 2.2%). The least coaggregation values with *S. aureus* and *B. cereus* were obtained for isolates F18 (2.5 ± 1.2) and F19 (1.4 ± 0.9).

### Adhesion assay

Based on the findings illustrated in Table 3, only five isolates (F3, F7, F8, F12, and F15) were able to adhere to Caco-2 cells. The adhesion values ranged from 0 to 38.7 ± 1.5%. Isolate F12 showed the highest ability to adhere to epithelial cells, followed by isolates F8 (31.4 ± 2.1%), F7 (27.5 ± 1.9%), F3 (24.7 ± 1.7%), and F15 (23.2 ± 1.55).

### Cholesterol removal

Table 3 illustrates the cholesterol assimilation patterns of the selected isolates. Cholesterol assimilation values varied among the 19 evaluated isolates and ranged from 3.6 to 78.2%. Isolate F12 showed the highest removal cholesterol ability, with value of 78.2%, followed by isolates F3 and F7, with values of 64.5% and 59.7%, respectively. Isolates F18 showed the lowest cholesterol assimilation (3.6%), followed by isolates F19 and F11, with values of 7.25% and 11.8%.

### Biofilm formation

**Figure 3:**
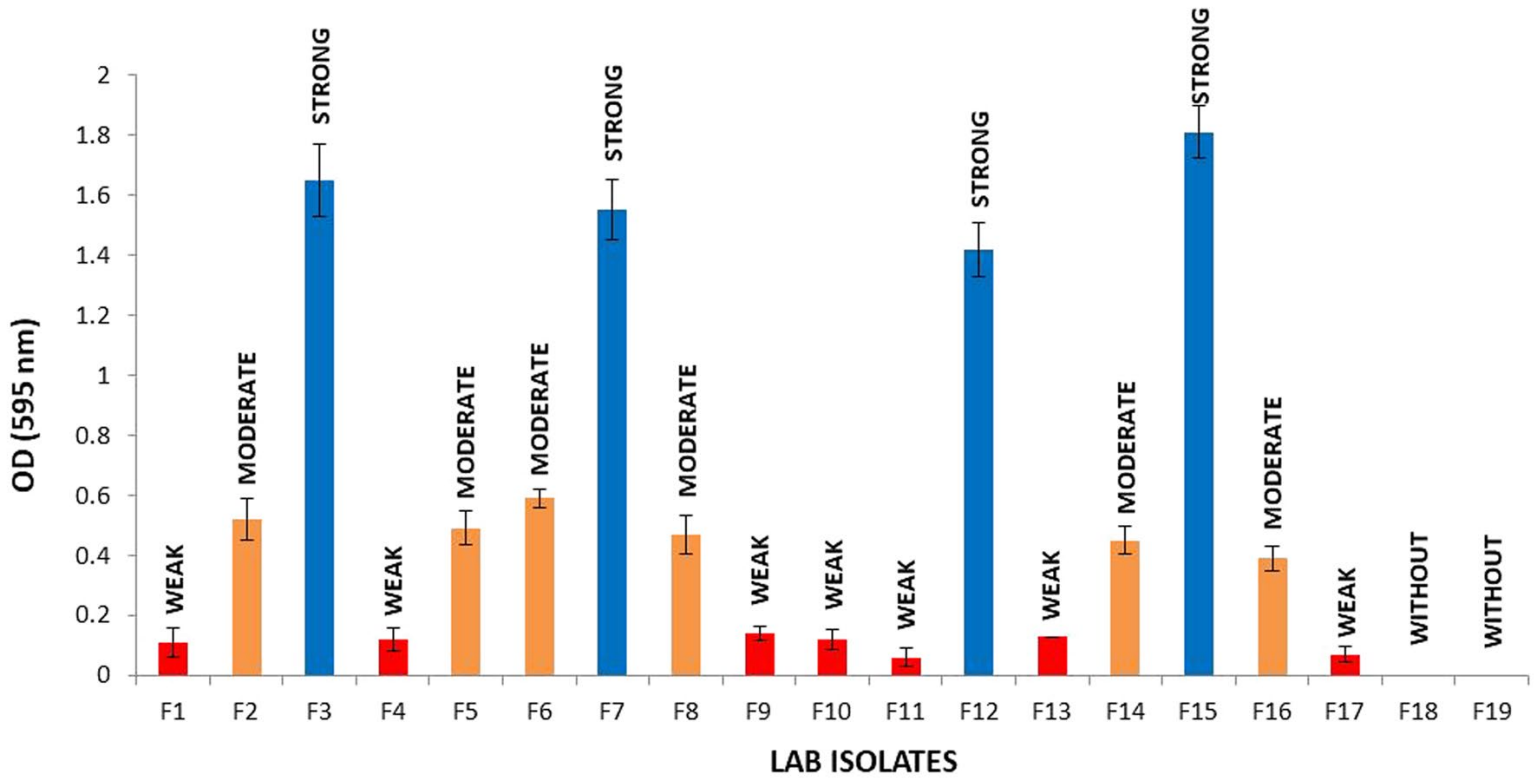
Biofilm formation ability of selected strains. Bars represent standard errors of the mean (n = 3). Based on the OD, bacteria were classified as non-biofilm producers (OD ≤ ODC), weak (ODC < OD ≤ 2 × ODC), moderate (2 × ODC < OD ≤ 4 × ODC) or strong biofilm producers (4 × ODC < OD; Borges et al., 2012). Where the cut-off (ODC) was defined as the mean OD value of the negative control.

The ability of isolates to form biofilm in MRS medium was illustrated in Fig. 3. Results showed that 17 out of 19 isolates were able to form biofilm. The highest biofilm formation values were observed for isolate F15, with OD_600_ = 1.81, followed by isolates F3 (OD_600_ = 1.65), F7 (OD_600_ = 1.55), and F12 (OD_600_ = 1.42). Four out of 17 isolates were strong biofilm producers, 6 isolates were moderate biofilm producers and 7 isolates were weak biofilm producers.

### Exopolysaccharide (EPS) production

The results showed that only six isolates (F3, F5, F7, F9, F12, and F15) were able to produce EPS. The highest values of EPS production were observed for isolate F3, followed by F15, F7, and F12.

### Molecular identification

The 16S-rRNA gene sequencing was used to validate the phenotypic characterization of the selected LAB isolates. The PCR-amplified 1544 bp fragments of the 16S-rRNA gene (Fig. 1) of the isolates were sequenced and blasted with the sequences deposited in GeneBank. Amplification of the 16S-rRNA genes of 19 LAB isolates confirmed that all isolates belonged to the 2 genera of LAB including *Lactobacillus*, and *Pediococcus*. *Lactobacillus* isolates classified to 4 species including *L. plantarum* (F1, F6, F8, F10, F11, F12), *L. acidophilus* (F3, F5), *L. brevis* (F17, F19), and *L. pentosus* (F16, F18). *Pediococcus* isolates classified to 2 species including *P. pentosaceus* (F7, F13, F14), and *P. acidilactici* (F2, F4, F9, F15).

### Clustering analysis using heat map and PCA methods

**Figure 4:**
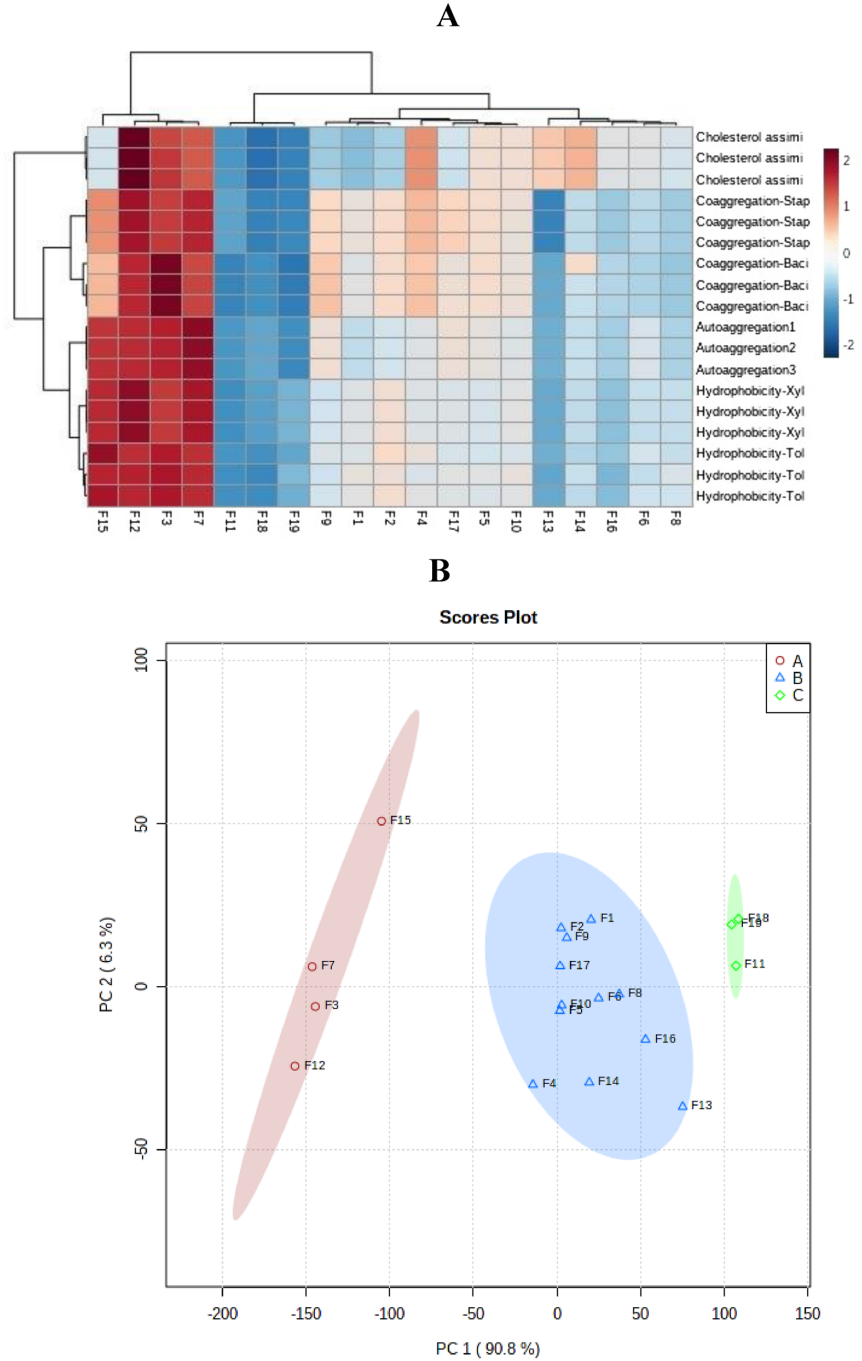
Cluster analysis of isolates based on probiotic characteristics using heat-map (**A**) and PCA (**B**) methods.

Identified and characterized strains with desired probiotic features such as acid and bile tolerance, cell surface hydrophobicity, auto-aggregation and co-aggregation were subjected to cluster analysis based on unsupervised methods. Unsupervised methods are types of learning algorithm used to knowledge discovery from datasets that are neither classified nor labeled^35–37^. As depicted in Fig. 4, Identified strains clustered in three distinct groups by Ward and PCA methods. It is obvious cluster I in heat map included the isolates F3, F7, F12, and F15, whereas; remaining nine strains created the other two clades. The projection of identified strains diversity based on the PC1 and PC2 highlighted the diversity of identified bacteria. In total, about 97% of the variation in the characterized bacteria was explained by two principal components (Table 3). More interestingly, congruence was displayed between the results of two applied methods in clustering the identified bacteria based on probiotic characteristics.

## Discussion

There are several criteria for bacteria to recognize as probiotic. One of the most important features is tolerance to low pH and high bile salt concentrations^6^. They must overcome physical and chemical barriers in the gastrointestinal tract to be able to transit through the stomach. Probiotics to be able to survive and grow and could exert their action in the small intestine must be tolerant to high bile salt conditions^38^. In this work, the evaluated isolates indicated varying values of viability in pH 3 and 0.3% bile oxgall. The isolates F3, F12, F15, and F7 showed more than 90% viability at pH 3 and 0.3% bile oxgall. High survival rates of LAB especially *L. plantarum*^24,39^, and *P. pentosaceus* and *P. acidilactici*^40–42^ at gastric pH of 3.0 and 0.3% bile concentration have been reported.

The antimicrobial properties of probiotics are attributed to their metabolite productions including organic acids, hydrogen peroxide, and bacteriocin^43^. In this study, isolates were investigated against two Gran-positive (*St. iniae* and *St. agalactia*) and six Gram-negative (*V. harveyi*, *V. cholera*, *V. alginolyticus*, *V. parahaemolyticus*, and C*l. cochlearium*) indicators. Evaluated isolates showed better antibacterial effects on Gram-positive than Gram-negative indicators, which this could be due to the outer membrane of Gram-negative bacteria. All the 19 isolates inhibited the growth of *St. iniae* and *St. agalactia.* Isolates F12 and F15 showed the best results and were able to inhibit the growth of all the 8 evaluated indicators.

Another strange property of probiotics is their ability to form biofilms. It can promote colonization within the inner wall of the intestine and longer lasting quality of LAB, thus maintaining a strategic distance from colonization by pathogenic microorganisms^44^. The ability of form biofilm is of great economic importance to food-processing companies, as subsequent microorganisms attached to industrial devices can form highly resistant biofilms, and their strong tendency to adhere to the surface facilitates the separation of biomass during the fermentation process^45^. Isolates F3, F7, F12, and F15 showed the strong biofilm formation ability; cause them to be suitable for use as biocontrol agents.

There are many studies, which investigated the cholesterol reduction ability of LAB isolates^28,46,47^. The reduction of serum cholesterol values is important to prevent the coronary heart disease due to the main risk factor for the progression of coronary heart disease is hypercholesterolemia^48^. Isolates F12, F3, and F7 showed remarkable cholesterol removal ability, with values of more than 59%.

For selection of functional LAB isolates, two groups of antibiotics are recommended; first those are inhibitors of cell wall synthesis such as vancomycin and ampicillin, and second those are inhibitors of protein synthesis including erythromycin, chloramphenicol, clindamycin, gentamycin, kanamycin, tetracycline, and streptomycin^49^. In this study, the LAB isolates indicated susceptibility to chloramphenicol (89.47%), tetracycline (94.73%), erythromycin (84.21%), ampicillin (84.21%), azithromycin (52.63%), gentamycin (42.11%), streptomycin (57.89%), penicillin (42.11%), vancomycin (52.63%), and ciprofloxacin (42.11%). The results of this study is consistent with the previous results which showed that LAB are susceptible to antibiotics inhibiting the proteins synthesis, such as chloramphenicol, tetracycline, and erythromycin, and are resistance towards glycopepticides antibiotics such as vancomycin^50^, and aminoglycosides antibiotics including streptomycin, kanamycin, and gentamycin^51^. Several LAB species belonged to especially *Lactobacillus*, *Pediococcus* and *Leuconostoc* have been reported to exhibit high levels of natural resistance to vancomycin, which is useful to separate them from other Gram-positive bacteria^41^.

A desirable property of probiotic bacteria is colonization in intestinal wall^28^. Hydrophobicity, auto-aggregation and adhesion to epithelial cells could help probiotics to colonize in intestinal cells. The hydrophobicity of the bacterial cell surface is one of the most important factors for probiotics to be colonized in human and animal gastrointestinal tracts. In this study, the cell surface hydrophobicity percentages of the isolates ranged from 3.7 ± 1.6 to 70.2 ± 2.0% with toluene and 2.2 ± 1.6% to 76.4 ± 2.1% with xylene. This result confirms the results of several previous researches reported by^52–54^. Four out of 19 isolates showed both considerable cell surface hydrophobicity and high auto-aggregation (> 67%), which indicated good colonization potential^55^. According to Vallejo et al. (2008), strains with values of autoaggregation higher than 65% are strongly autoaggregating. Isolates F3, F7, F12, and F15 showed remarkable autoaggregation ability; making these isolates interesting candidates for potential probiotic applications. The capacity to attach to epithelial cells and mucosal surfaces may be a critical feature of bacterial isolates to be utilized as probiotics. Results showed that only isolates F3, F7, F8, F12, and F15 were able to adhere to Caco-2 cells. The adhesion ability of these 4 isolates to Caco-2 cell had also significant coherence to auto-aggregation and hydrophobicity. The adhesion rates of these isolates to Caco-2 cells were between 23 and 38%. It seems that adhesion ability is correlated with auto-aggregation^46^. These results also support the correlation between adhesion and auto-aggregation.

In conclusion, several isolates evaluated in this study showed potential probiotic properties. The majority of evaluated isolates showed high antimicrobial activities against potentially pathogenic Gram-positive and Gram-negative indicator bacteria, which deserve them to be used as biocontrol agents in fishery. The highest values of autoaggregation and hydrophobicity as well as a high value of coaggregation and a high capacity for biofilm formation, isolates F3, F7, F12, and F15 could be candidates to be used as bio-control agents in fishery to protect against pathogenic microorganisms and improve health care and food safety by avoiding the use of additives. Cluster characterization of identified potential probiotic bacteria based on two above-mentioned methods also highlighted the priority of isolates F3, F7, F12, and F15 as bio-control agents in fishery industry. In line with our study, prior finding also highlighted the effectiveness of above-mentioned methods for characterization and prioritization of newly identified isolates^33,56^. In addition, our empirical and cluster analysis based on unsupervised methods prioritized some isolates as biocontrol agent candidates in fishery industry, however; further analysis should be performed.

## Data Availability

The datasets generated during the current study are available in the NCBI repository, *L. acidophilus* GS1 (Accession No. ON210277); *L. acidophilus* GS2 (Accession No. ON210276); *P. acidilactici* GS3 (Accession No. ON210279); *P. acidilactici* GS4 (Accession No. ON210280); *P. acidilactici* GS5 (Accession No. ON210274); *P. acidilactici* GS6 (Accession No. ON210278); *P. pentosaceus* GS7 (Accession No. ON210281); *P. pentosaceus* GS8 (Accession No. ON210275); *P. pentosaceus* GS9 (Accession No. ON210282); *L. brevis* GS-10 (Accession No. ON248836); *L. brevis* GS-11 (Accession No. ON248846); *L. plantarum* GS-12 (Accession No. ON248840); *L. plantarum* GS-13 (Accession No. ON248843); *L. plantarum* GS-14 (Accession No. ON248839); *L. plantarum* GS-15 (Accession No. ON248848); *L. plantarum* GS-16 (Accession No. ON248847); *L. plantarum* GS-17 (Accession No. ON248850); *L. pentosus* GS-18 (Accession No. ON248844); *L. pentosus* GS-19 (Accession No. ON248841).
